# 6,7,6′,7′-Tetra­phenyl-2,2′-bi[1,3-dithia-5,8-diaza­cyclo­penta­[*b*]naphthalenyl­idene] chloro­form disolvate

**DOI:** 10.1107/S1600536811029540

**Published:** 2011-08-02

**Authors:** Ramababu Bolligarla, Gummadi Durgaprasad, Samar K. Das

**Affiliations:** a School of Chemistry, Univerisity of Hyderabad, Hyderabad 500 046, India

## Abstract

The title compound, C_42_H_24_N_4_S_4_·2CHCl_3_, a symmetrical tetra­thia­fulvalene (TTF) derivative, was prepared by a tri­ethyl­phos­phite-mediated self-coupling reaction of 6,7-diphenyl-1,3-dithia-5,8-diaza­cyclo­penta­[*b*]napthalen-2-one. The asymmetric unit contains two TTF mol­ecules and four chloro­form solvent mol­ecules. Cl⋯Cl inter­actions [contact distances = 3.263 (1)–3.395 (2) Å] are present between the solvent mol­ecules, resulting in a tape along the *bc* plane. The crystal packing features weak C—H⋯Cl and C—H⋯N hydrogen bonds, resulting in the formation of a two-dimensional supramolecular network.

## Related literature

For TTF chemistry, see: Bendikov *et al.* (2004 ▶). For conductors and super-conductors, see: Yamada *et al.* (2004 ▶); Otsubo & Takimiya (2004 ▶). For field effect transistors, see: Mas-Torrent *et al.* (2004 ▶); Noda *et al.* (2005 ▶); Naraso *et al.* (2005 ▶). For the synthesis see: Bolligarla & Das (2011 ▶). For bond lengths in TTF derivatives, see: Bouguessa *et al.* (2003 ▶). 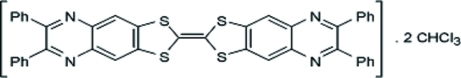


## Experimental

### 

#### Crystal data

C_42_H_24_N_4_S_4_·2CHCl_3_
*M* *_r_* = 951.63
Monoclinic, 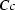

*a* = 14.5359 (11) Å
*b* = 14.7543 (11) Å
*c* = 39.771 (3) Å
β = 97.616 (2)°
*V* = 8454.3 (11) Å^3^
*Z* = 8
Mo *K*α radiation
μ = 0.64 mm^−1^
*T* = 100 K
0.48 × 0.36 × 0.14 mm

#### Data collection

Bruker SMART CCD area-detector diffractometer
Absorption correction: multi-scan (*SADABS*; Sheldrick, 1996 ▶) *T*_min_ = 0.748, *T*_max_ = 0.915
43023 measured reflections
16629 independent reflections
16092 reflections with *I* > 2σ(*I*)
*R*_int_ = 0.029

#### Refinement

*R*[*F* ^2^ > 2σ(*F* ^2^)] = 0.042
*wR*(*F* ^2^) = 0.109
*S* = 1.07
16629 reflections
1045 parameters
2 restraints
H-atom parameters constrained
Δρ_max_ = 0.58 e Å^−3^
Δρ_min_ = −0.66 e Å^−3^

### 

Data collection: *SMART* (Bruker, 2002 ▶); cell refinement: *SAINT* (Bruker, 2002 ▶); data reduction: *SAINT*; program(s) used to solve structure: *SHELXS97* (Sheldrick, 2008 ▶); program(s) used to refine structure: *SHELXL97* (Sheldrick, 2008 ▶); molecular graphics: *SHELXTL* (Sheldrick, 2008 ▶); software used to prepare material for publication: *SHELXTL*.

## Supplementary Material

Crystal structure: contains datablock(s) I, global. DOI: 10.1107/S1600536811029540/ds2113sup1.cif

Structure factors: contains datablock(s) I. DOI: 10.1107/S1600536811029540/ds2113Isup2.hkl

Supplementary material file. DOI: 10.1107/S1600536811029540/ds2113Isup3.cml

Additional supplementary materials:  crystallographic information; 3D view; checkCIF report

## Figures and Tables

**Table 1:** Hydrogen-bond geometry (Å, °)

*D*—H⋯*A*	*D*—H	H⋯*A*	*D*⋯*A*	*D*—H⋯*A*
C8—H8⋯Cl2^i^	0.93	2.94	3.676 (4)	137
C85—H85⋯N2^ii^	0.98	2.31	3.233 (5)	156
C12—H12⋯N6^iii^	0.93	2.61	3.344 (4)	136
C86—H86⋯N3^iv^	0.98	2.29	3.223 (5)	158
C88—H88⋯N8^iv^	0.98	2.28	3.199 (5)	155
C87—H87⋯N5^v^	0.98	2.32	3.246 (5)	157
C60—H60⋯N1^vi^	0.93	2.63	3.392 (5)	139
C78—H78⋯N4^vii^	0.93	2.62	3.427 (4)	145
C42—H42⋯N7^v^	0.93	2.61	3.358 (4)	138

Symmetry codes: (i) 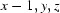; (ii) 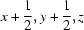; (iii) 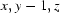; (iv) 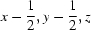; (v) 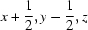; (vi) 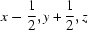; (vii) 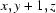.

**Fig. 1.**
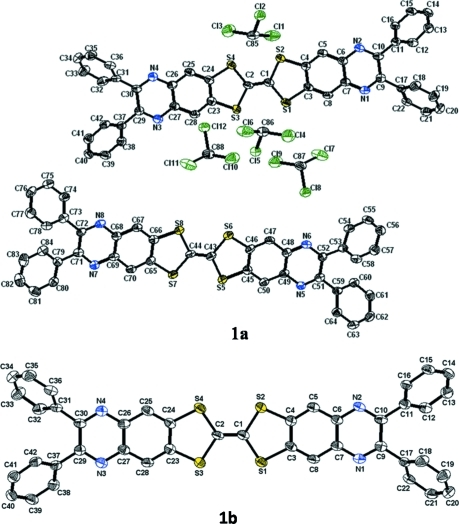
(a)Thermal ellipsoidal plot of the asymmetric unit of compound, the asymmetric unit contain two units of two TTF molecules and four chloroform solvent molecules. Hydrogen atoms are not shown for clarity (70% probability); (b)Thermal ellipsoidal plot of one of the molecules present in the asymmetric unit of the compound, Hydrogen atoms are not shown for clarity (70% probability).

**Fig. 2.**
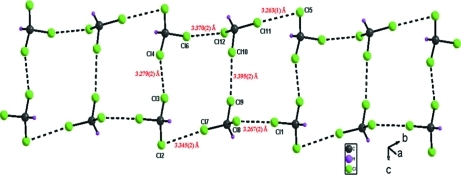
The Cl···Cl interactions are between the chloroform solvent molecules to form a one dimensional tape.

**Fig. 3.**
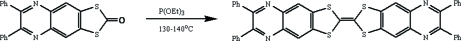
The formation of the title compound.

**Fig. 4.**
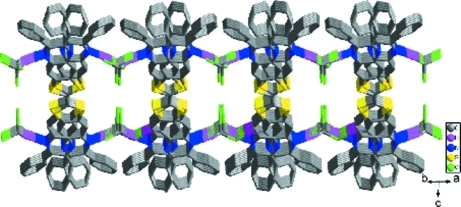
Crystal packing diagram of the compound is described by C—H···N and C—H···Cl weak interactions resulting in two dimensional supramolecular network.

## supplementary crystallographic information

### Comment

Research interests on tetrathiafulvalene (TTF)–based compounds have remained dynamic in the field of materials science, particularly, in the context of molecular electronics and NLO materials, due to their unique π-donor properties. TTF and its derivatives have successfully been used as versatile building blocks for the formation of charge transfer salts giving rise to organic conductors and even super-conductors [see: Yamada *et al.* (2004); Otsubo *et al.* (2004)]. Furthermore, tetrathiafulvalene (TTF) derivatives are promising candidates for semiconductors leading to high performance FETs (Field Effect Transistors) because of their self-assembling properties. However, because of the strong electron-donating properties, the relevant thin films are generally labile to oxygen, resulting in poor FET performance. Naraso *et al.* have introduced fused aromatic rings or electron-deficient nitrogen heterocycles to the TTF skeleton to enhance the stability and obtained high hole mobilities in the thin films. In our previous letter [Bolligarla *et al.* (2011)], we have reported the synthesis and physical properties of acceptor-donor-acceptor (A—D—A) TTF (title compound). In solution state, emission behavior of this compound has also been described which is largely solvent dependent with huge Stokes shifts. In this contribution, we have reported the crystal structure and supramolecular feature of the title compound. The asymmetric unit contains two molecules of TTF triad and four molecules of chloroform (solvent) molecules as shown in Fig. 1(*a*). For clarity, one of the molecules present in the asymmetric unit is shown in Fig. 1(*b*). As shown in Fig. 1(*b*), the skeleton of the molecule is almost planar excluding the four peripheral phenyl groups. The r.m.s. deviation from a least-squares plane through the atoms of the core is 0.027 Å. The phenyl rings are deviated from the plane of skeleton of the molecule with angles in the range from 36.03° to 55.81°. The bond lengths in the TTF moiety are in the range of bond lengths, expected for neutral TTF derivatives. Interestingly, six Cl···Cl interactions are present between the solvent molecules resulting in the formation of a one dimensional chloroform tapes, and the Cl···Cl intermolecular contact distances are in the range from 3.263 (1) to 3. 395 (2) Å as shown in Fig. 2.

### Experimental

The title compound was synthesized according to literature procedure [Bolligarla *et al.* (2011)]. A solution of compound 6,7-diphenyl-1,3-dithia-5,8-diaza-cyclopenta[*b*]napthalen-2-one (125 mg, 0.336 mmol) in triethylphosphite (3 mL) was refluxed at 130–140 °C for 2 h under N_2_ atmosphere. After cooling to room temperature, MeOH (20 ml) was added and the resulting orange precipitate was filtered off (Yield: 70.0%). Single crystals of title compound, suitable for single-crystal X-ray analysis was obtained from chloroform in an NMR tube on slow evaporation over a period of two weeks.

### Refinement

All non-hydrogen atoms was refined anisotropically. The hydrogen atoms were included in the structure factor calculation by using a riding model.

### Crystal data

**Table:**

C_42_H_24_N_4_S_4_·2CHCl_3_	*F*(000) = 3872
*M**_r_* = 951.63	*D*_x_ = 1.495 Mg m^−^^3^
Monoclinic, *C**c*	Mo *K*α radiation, λ = 0.71073 Å
Hall symbol: C -2yc	Cell parameters from 8772 reflections
*a* = 14.5359 (11) Å	θ = 2.3–26.2°
*b* = 14.7543 (11) Å	µ = 0.64 mm^−^^1^
*c* = 39.771 (3) Å	*T* = 100 K
β = 97.616 (2)°	Block, brown
*V* = 8454.3 (11) Å^3^	0.48 × 0.36 × 0.14 mm
*Z* = 8	

### Data collection

**Table:**

Bruker SMART CCD area-detector diffractometer	16629 independent reflections
Radiation source: fine-focus sealed tube	16092 reflections with *I* > 2σ(*I*)
graphite	*R*_int_ = 0.029
φ and ω scans	θ_max_ = 26.2°, θ_min_ = 2.0°
Absorption correction: multi-scan (*SADABS*; Sheldrick, 1996)	*h* = −18→17
*T*_min_ = 0.748, *T*_max_ = 0.915	*k* = −18→18
43023 measured reflections	*l* = −48→49

### Refinement

**Table:**

Refinement on *F*^2^	Primary atom site location: structure-invariant direct methods
Least-squares matrix: full	Secondary atom site location: difference Fourier map
*R*[*F*^2^ > 2σ(*F*^2^)] = 0.042	Hydrogen site location: inferred from neighbouring sites
*wR*(*F*^2^) = 0.109	H-atom parameters constrained
*S* = 1.07	*w* = 1/[σ^2^(*F*_o_^2^) + (0.0576*P*)^2^ + 12.7425*P*] where *P* = (*F*_o_^2^ + 2*F*_c_^2^)/3
16629 reflections	(Δ/σ)_max_ = 0.001
1045 parameters	Δρ_max_ = 0.58 e Å^−^^3^
2 restraints	Δρ_min_ = −0.66 e Å^−^^3^

### Special details

**Table:**

Geometry. All e.s.d.'s (except the e.s.d. in the dihedral angle between two l.s. planes) are estimated using the full covariance matrix. The cell e.s.d.'s are taken into account individually in the estimation of e.s.d.'s in distances, angles and torsion angles; correlations between e.s.d.'s in cell parameters are only used when they are defined by crystal symmetry. An approximate (isotropic) treatment of cell e.s.d.'s is used for estimating e.s.d.'s involving l.s. planes.
Refinement. Refinement of *F*^2^ against ALL reflections. The weighted *R*-factor *wR* and goodness of fit *S* are based on *F*^2^, conventional *R*-factors *R* are based on *F*, with *F* set to zero for negative *F*^2^. The threshold expression of *F*^2^ > σ(*F*^2^) is used only for calculating *R*-factors(gt) *etc*. and is not relevant to the choice of reflections for refinement. *R*-factors based on *F*^2^ are statistically about twice as large as those based on *F*, and *R*- factors based on ALL data will be even larger.Absolute structure of the title compound could not be determined unambigously due to the lack of enough contribution towards anomalous dispersion by the non hydrogen atoms present and therefore, the Flack parameter is not reported.

### Fractional atomic coordinates and isotropic or equivalent isotropic displacement parameters (Å^2^)

**Table:**

	*x*	*y*	*z*	*U*_iso_*/*U*_eq_	
S5	0.12002 (6)	0.93290 (6)	0.57016 (2)	0.02468 (18)	
S4	0.58994 (6)	0.14264 (6)	0.51422 (2)	0.02435 (18)	
S8	0.35756 (6)	0.86813 (6)	0.51766 (2)	0.02512 (18)	
S1	0.37317 (6)	0.14711 (6)	0.58085 (2)	0.02433 (18)	
S7	0.22618 (6)	1.02323 (6)	0.51246 (2)	0.02470 (18)	
S3	0.44035 (6)	0.27438 (6)	0.52303 (2)	0.02481 (18)	
S6	0.25227 (6)	0.77850 (6)	0.57503 (2)	0.02452 (18)	
S2	0.52343 (6)	0.01627 (6)	0.57179 (2)	0.02431 (18)	
C68	0.4590 (2)	1.0229 (2)	0.44690 (8)	0.0207 (7)	
N7	0.39830 (19)	1.16091 (19)	0.42016 (7)	0.0210 (6)	
N3	0.5599 (2)	0.4788 (2)	0.43348 (7)	0.0232 (6)	
C17	0.2316 (2)	−0.1369 (2)	0.72103 (8)	0.0195 (7)	
C31	0.7318 (2)	0.4258 (2)	0.37375 (8)	0.0209 (7)	
N6	0.08049 (19)	0.63969 (19)	0.66733 (7)	0.0208 (6)	
C26	0.6392 (2)	0.3373 (2)	0.44750 (8)	0.0207 (7)	
N2	0.40713 (19)	−0.18868 (19)	0.66189 (7)	0.0207 (6)	
C7	0.3249 (2)	−0.0484 (2)	0.64744 (8)	0.0203 (7)	
N4	0.69477 (19)	0.34997 (19)	0.42295 (7)	0.0205 (6)	
C80	0.4410 (2)	1.3117 (2)	0.37982 (8)	0.0231 (7)	
H80	0.4365	1.3281	0.4021	0.028*	
N5	−0.0499 (2)	0.78039 (19)	0.66059 (7)	0.0201 (6)	
C49	0.0203 (2)	0.7780 (2)	0.64082 (8)	0.0208 (7)	
C27	0.5694 (2)	0.4015 (2)	0.45210 (8)	0.0208 (7)	
C3	0.3766 (2)	0.0475 (2)	0.60497 (8)	0.0204 (7)	
C72	0.5312 (2)	1.0836 (2)	0.40408 (8)	0.0205 (7)	
C53	0.0259 (2)	0.5814 (2)	0.71666 (8)	0.0198 (7)	
N8	0.52848 (19)	1.0185 (2)	0.42723 (7)	0.0217 (6)	
C70	0.3237 (2)	1.0982 (2)	0.46466 (8)	0.0220 (7)	
H70	0.2830	1.1471	0.4633	0.026*	
C79	0.4564 (2)	1.2221 (2)	0.37206 (8)	0.0206 (7)	
N1	0.27045 (19)	−0.06132 (19)	0.67222 (7)	0.0217 (6)	
C48	0.0841 (2)	0.7047 (2)	0.64326 (8)	0.0213 (7)	
C5	0.4564 (2)	−0.0944 (2)	0.61861 (8)	0.0214 (7)	
H5	0.5013	−0.1366	0.6147	0.026*	
C23	0.5161 (2)	0.3050 (2)	0.49429 (8)	0.0215 (7)	
C6	0.3965 (2)	−0.1118 (2)	0.64298 (8)	0.0195 (7)	
C52	0.0166 (2)	0.6463 (2)	0.68797 (8)	0.0195 (7)	
C46	0.1639 (2)	0.7729 (2)	0.60089 (8)	0.0200 (7)	
C71	0.4624 (2)	1.1536 (2)	0.39973 (8)	0.0206 (7)	
C63	−0.2366 (3)	0.8073 (3)	0.73290 (9)	0.0287 (8)	
H63	−0.2555	0.8629	0.7406	0.034*	
C37	0.6026 (2)	0.5772 (2)	0.39069 (8)	0.0211 (7)	
C73	0.6101 (2)	1.0780 (2)	0.38364 (8)	0.0232 (7)	
C43	0.2165 (2)	0.8821 (2)	0.55573 (8)	0.0225 (7)	
C47	0.1552 (2)	0.7027 (2)	0.62264 (8)	0.0220 (7)	
H47	0.1962	0.6540	0.6238	0.026*	
C16	0.4548 (2)	−0.3216 (2)	0.71374 (8)	0.0205 (7)	
H16	0.5061	−0.2893	0.7086	0.025*	
C4	0.4483 (2)	−0.0150 (2)	0.60068 (8)	0.0194 (6)	
C75	0.7122 (3)	0.9879 (3)	0.35420 (10)	0.0292 (8)	
H75	0.7312	0.9315	0.3473	0.035*	
C64	−0.1626 (2)	0.8035 (2)	0.71444 (9)	0.0244 (7)	
H64	−0.1332	0.8566	0.7092	0.029*	
C8	0.3153 (2)	0.0312 (2)	0.62766 (8)	0.0216 (7)	
H8	0.2679	0.0722	0.6300	0.026*	
C59	−0.1318 (2)	0.7194 (2)	0.70355 (8)	0.0198 (7)	
C22	0.1364 (2)	−0.1222 (2)	0.71611 (9)	0.0228 (7)	
H22	0.1063	−0.1097	0.6945	0.027*	
C36	0.8284 (3)	0.4120 (2)	0.37773 (9)	0.0256 (7)	
H36	0.8605	0.4017	0.3992	0.031*	
C24	0.5876 (2)	0.2414 (2)	0.49009 (8)	0.0205 (7)	
C65	0.3148 (2)	1.0287 (2)	0.48684 (8)	0.0207 (7)	
C45	0.1000 (2)	0.8464 (2)	0.59820 (8)	0.0215 (7)	
C18	0.2760 (3)	−0.1517 (3)	0.75389 (9)	0.0289 (8)	
H18	0.3399	−0.1602	0.7575	0.035*	
C69	0.3949 (2)	1.0949 (2)	0.44409 (9)	0.0216 (7)	
C11	0.3653 (2)	−0.2869 (2)	0.70489 (8)	0.0211 (7)	
C25	0.6484 (2)	0.2580 (2)	0.46729 (8)	0.0220 (7)	
H25	0.6957	0.2169	0.4649	0.026*	
C67	0.4498 (2)	0.9523 (2)	0.47032 (8)	0.0225 (7)	
H67	0.4923	0.9049	0.4727	0.027*	
C28	0.5076 (2)	0.3840 (2)	0.47621 (8)	0.0219 (7)	
H28	0.4618	0.4258	0.4796	0.026*	
C10	0.3537 (2)	−0.2001 (2)	0.68596 (8)	0.0210 (7)	
C2	0.4958 (2)	0.1718 (2)	0.53564 (8)	0.0217 (7)	
C74	0.6393 (2)	0.9941 (2)	0.37293 (9)	0.0251 (7)	
H74	0.6092	0.9417	0.3785	0.030*	
C51	−0.0528 (2)	0.7167 (2)	0.68359 (8)	0.0208 (7)	
C1	0.4669 (2)	0.1187 (2)	0.55954 (8)	0.0225 (7)	
C38	0.5142 (2)	0.6136 (2)	0.38140 (9)	0.0252 (7)	
H38	0.4622	0.5831	0.3868	0.030*	
C30	0.6805 (2)	0.4221 (2)	0.40339 (8)	0.0195 (7)	
C78	0.6585 (2)	1.1555 (2)	0.37596 (9)	0.0242 (7)	
H78	0.6414	1.2117	0.3837	0.029*	
C61	−0.2542 (3)	0.6465 (3)	0.72855 (9)	0.0273 (8)	
H61	−0.2862	0.5941	0.7329	0.033*	
C29	0.6135 (2)	0.4903 (2)	0.40974 (8)	0.0199 (7)	
C60	−0.1785 (2)	0.6410 (2)	0.71077 (8)	0.0227 (7)	
H60	−0.1591	0.5849	0.7037	0.027*	
C41	0.6691 (3)	0.7090 (3)	0.36691 (9)	0.0266 (8)	
H41	0.7210	0.7417	0.3627	0.032*	
C54	0.0349 (2)	0.4884 (2)	0.71084 (9)	0.0246 (7)	
H54	0.0318	0.4666	0.6888	0.030*	
C50	0.0281 (2)	0.8484 (2)	0.61736 (8)	0.0214 (7)	
H50	−0.0148	0.8955	0.6149	0.026*	
C66	0.3785 (2)	0.9539 (2)	0.48946 (8)	0.0214 (7)	
C9	0.2856 (2)	−0.1324 (2)	0.69186 (8)	0.0213 (7)	
C40	0.5808 (3)	0.7428 (2)	0.35680 (9)	0.0264 (7)	
H40	0.5729	0.7974	0.3451	0.032*	
C21	0.0860 (3)	−0.1262 (2)	0.74348 (10)	0.0271 (8)	
H21	0.0220	−0.1179	0.7400	0.032*	
C58	0.0324 (3)	0.6119 (2)	0.75016 (9)	0.0269 (7)	
H58	0.0258	0.6733	0.7545	0.032*	
C33	0.7330 (3)	0.4383 (3)	0.31369 (10)	0.0328 (8)	
H33	0.7008	0.4460	0.2921	0.039*	
C14	0.3904 (3)	−0.4529 (2)	0.73799 (9)	0.0263 (7)	
H14	0.3986	−0.5083	0.7491	0.032*	
C44	0.2612 (2)	0.9198 (2)	0.53193 (8)	0.0216 (7)	
C81	0.4321 (3)	1.3774 (3)	0.35454 (10)	0.0320 (8)	
H81	0.4211	1.4375	0.3599	0.038*	
C77	0.7313 (3)	1.1497 (3)	0.35712 (10)	0.0308 (8)	
H77	0.7629	1.2017	0.3521	0.037*	
C42	0.6796 (2)	0.6263 (2)	0.38336 (8)	0.0226 (7)	
H42	0.7388	0.6030	0.3896	0.027*	
C13	0.3022 (3)	−0.4186 (2)	0.72916 (9)	0.0265 (7)	
H13	0.2510	−0.4514	0.7341	0.032*	
C32	0.6861 (2)	0.4391 (3)	0.34140 (9)	0.0274 (8)	
H32	0.6223	0.4489	0.3384	0.033*	
C55	0.0485 (2)	0.4289 (2)	0.73784 (10)	0.0284 (8)	
H55	0.0524	0.3671	0.7337	0.034*	
C39	0.5040 (3)	0.6944 (2)	0.36430 (9)	0.0280 (8)	
H39	0.4447	0.7171	0.3576	0.034*	
C76	0.7571 (2)	1.0655 (3)	0.34570 (9)	0.0302 (8)	
H76	0.8048	1.0614	0.3323	0.036*	
C35	0.8754 (3)	0.4136 (3)	0.34986 (10)	0.0315 (8)	
H35	0.9395	0.4063	0.3527	0.038*	
C15	0.4667 (2)	−0.4050 (2)	0.73035 (9)	0.0250 (7)	
H15	0.5261	−0.4283	0.7363	0.030*	
C62	−0.2825 (3)	0.7294 (3)	0.73994 (9)	0.0292 (8)	
H62	−0.3325	0.7324	0.7523	0.035*	
C34	0.8282 (3)	0.4260 (3)	0.31783 (10)	0.0322 (8)	
H34	0.8602	0.4261	0.2991	0.039*	
C56	0.0565 (2)	0.4594 (3)	0.77062 (10)	0.0296 (8)	
H56	0.0671	0.4188	0.7886	0.036*	
C19	0.2261 (3)	−0.1538 (3)	0.78084 (9)	0.0325 (8)	
H19	0.2567	−0.1629	0.8026	0.039*	
C57	0.0484 (3)	0.5518 (3)	0.77677 (10)	0.0303 (8)	
H57	0.0538	0.5730	0.7990	0.036*	
C83	0.4533 (3)	1.2638 (3)	0.31343 (10)	0.0340 (9)	
H83	0.4572	1.2475	0.2911	0.041*	
C84	0.4615 (2)	1.1974 (3)	0.33861 (9)	0.0268 (7)	
H84	0.4703	1.1371	0.3331	0.032*	
C82	0.4396 (3)	1.3532 (3)	0.32132 (10)	0.0350 (9)	
H82	0.4354	1.3971	0.3044	0.042*	
C20	0.1307 (3)	−0.1424 (3)	0.77592 (9)	0.0295 (8)	
H20	0.0970	−0.1457	0.7942	0.035*	
C12	0.2895 (2)	−0.3356 (2)	0.71289 (9)	0.0244 (7)	
H12	0.2299	−0.3125	0.7073	0.029*	
Cl8	0.19602 (6)	0.45268 (6)	0.64885 (2)	0.03064 (19)	
Cl5	−0.10844 (6)	0.22596 (6)	0.44357 (2)	0.0317 (2)	
Cl12	0.27482 (6)	0.34015 (6)	0.44034 (2)	0.0313 (2)	
Cl3	0.99363 (8)	0.14803 (9)	0.58894 (3)	0.0480 (3)	
Cl4	−0.02752 (9)	0.13926 (9)	0.50589 (3)	0.0495 (3)	
Cl10	0.22187 (9)	0.41714 (9)	0.50180 (3)	0.0539 (3)	
Cl11	0.28527 (8)	0.53372 (7)	0.45119 (4)	0.0497 (3)	
Cl9	0.25430 (9)	0.38265 (9)	0.58694 (3)	0.0515 (3)	
Cl7	0.18825 (8)	0.26046 (7)	0.63526 (4)	0.0513 (3)	
C87	0.2487 (3)	0.3614 (3)	0.63006 (10)	0.0308 (8)	
H87	0.3122	0.3547	0.6416	0.037*	
C88	0.2241 (3)	0.4340 (3)	0.45824 (10)	0.0310 (8)	
H88	0.1601	0.4404	0.4472	0.037*	
Cl1	0.88234 (7)	0.06387 (8)	0.63569 (3)	0.0472 (3)	
Cl6	0.09042 (8)	0.22708 (8)	0.46238 (4)	0.0553 (3)	
C86	−0.0125 (3)	0.1644 (3)	0.46373 (10)	0.0332 (8)	
H86	−0.0078	0.1074	0.4515	0.040*	
C85	0.9839 (3)	0.1270 (3)	0.63160 (10)	0.0323 (8)	
H85	0.9802	0.1852	0.6432	0.039*	
Cl2	1.08202 (6)	0.06723 (6)	0.65138 (2)	0.03064 (19)	

### Atomic displacement parameters (Å^2^)

**Table:**

	*U*^11^	*U*^22^	*U*^33^	*U*^12^	*U*^13^	*U*^23^
S5	0.0254 (4)	0.0253 (4)	0.0246 (4)	0.0057 (3)	0.0081 (3)	0.0018 (3)
S4	0.0264 (4)	0.0197 (4)	0.0264 (4)	0.0062 (3)	0.0018 (3)	0.0033 (3)
S8	0.0252 (4)	0.0237 (4)	0.0281 (4)	0.0055 (3)	0.0095 (3)	0.0000 (3)
S1	0.0260 (4)	0.0205 (4)	0.0261 (4)	0.0069 (3)	0.0017 (3)	0.0028 (3)
S7	0.0236 (4)	0.0260 (4)	0.0257 (4)	0.0068 (3)	0.0075 (3)	−0.0012 (3)
S3	0.0269 (4)	0.0226 (4)	0.0253 (4)	0.0067 (3)	0.0050 (3)	0.0049 (3)
S6	0.0220 (4)	0.0259 (4)	0.0268 (4)	0.0045 (3)	0.0075 (3)	−0.0016 (3)
S2	0.0260 (4)	0.0200 (4)	0.0275 (4)	0.0060 (3)	0.0056 (3)	0.0044 (3)
C68	0.0197 (16)	0.0226 (17)	0.0187 (15)	0.0020 (13)	−0.0020 (12)	−0.0048 (12)
N7	0.0194 (14)	0.0232 (15)	0.0193 (14)	0.0027 (11)	−0.0013 (11)	−0.0024 (11)
N3	0.0265 (15)	0.0206 (14)	0.0216 (14)	0.0043 (12)	0.0000 (11)	−0.0013 (11)
C17	0.0227 (16)	0.0140 (15)	0.0219 (16)	0.0014 (12)	0.0030 (13)	−0.0022 (12)
C31	0.0218 (16)	0.0165 (16)	0.0242 (17)	0.0049 (13)	0.0019 (13)	−0.0044 (13)
N6	0.0208 (14)	0.0187 (14)	0.0221 (14)	0.0035 (11)	0.0000 (11)	−0.0028 (11)
C26	0.0206 (16)	0.0203 (16)	0.0206 (16)	0.0004 (13)	0.0007 (12)	−0.0064 (13)
N2	0.0204 (14)	0.0163 (13)	0.0245 (14)	0.0046 (11)	−0.0006 (11)	−0.0006 (11)
C7	0.0205 (16)	0.0200 (16)	0.0194 (15)	0.0028 (13)	−0.0013 (12)	−0.0038 (13)
N4	0.0201 (14)	0.0217 (14)	0.0187 (13)	0.0016 (11)	−0.0013 (11)	−0.0028 (11)
C80	0.0164 (15)	0.0318 (19)	0.0207 (16)	0.0022 (14)	0.0012 (12)	−0.0008 (14)
N5	0.0209 (14)	0.0196 (14)	0.0199 (13)	0.0025 (11)	0.0025 (11)	−0.0013 (11)
C49	0.0174 (15)	0.0261 (18)	0.0185 (15)	0.0052 (13)	0.0005 (12)	−0.0032 (13)
C27	0.0214 (16)	0.0194 (16)	0.0203 (16)	0.0037 (13)	−0.0020 (13)	−0.0017 (13)
C3	0.0254 (17)	0.0160 (16)	0.0179 (15)	0.0038 (13)	−0.0043 (13)	−0.0012 (12)
C72	0.0187 (16)	0.0251 (17)	0.0177 (15)	0.0004 (13)	0.0020 (12)	−0.0073 (13)
C53	0.0138 (15)	0.0217 (17)	0.0234 (16)	0.0037 (12)	0.0010 (12)	−0.0004 (13)
N8	0.0148 (13)	0.0258 (15)	0.0243 (14)	0.0029 (11)	0.0020 (11)	−0.0054 (12)
C70	0.0238 (17)	0.0216 (17)	0.0203 (16)	0.0053 (13)	0.0013 (13)	−0.0045 (13)
C79	0.0131 (15)	0.0232 (17)	0.0248 (16)	−0.0001 (13)	0.0001 (12)	0.0011 (13)
N1	0.0186 (14)	0.0193 (14)	0.0265 (15)	0.0030 (11)	0.0009 (11)	−0.0036 (11)
C48	0.0193 (16)	0.0243 (17)	0.0198 (16)	0.0011 (13)	0.0006 (12)	−0.0042 (13)
C5	0.0198 (16)	0.0194 (16)	0.0240 (17)	0.0047 (13)	−0.0005 (13)	−0.0019 (13)
C23	0.0237 (16)	0.0204 (17)	0.0193 (16)	0.0014 (13)	−0.0012 (13)	−0.0010 (12)
C6	0.0223 (16)	0.0154 (15)	0.0195 (16)	−0.0003 (13)	−0.0023 (13)	−0.0047 (12)
C52	0.0181 (15)	0.0221 (17)	0.0180 (15)	0.0005 (13)	0.0016 (12)	−0.0041 (12)
C46	0.0144 (15)	0.0285 (18)	0.0167 (15)	0.0029 (13)	0.0012 (12)	−0.0054 (13)
C71	0.0162 (15)	0.0222 (17)	0.0220 (16)	0.0005 (13)	−0.0030 (12)	−0.0049 (13)
C63	0.0259 (19)	0.028 (2)	0.032 (2)	0.0086 (15)	0.0062 (15)	−0.0042 (15)
C37	0.0234 (16)	0.0194 (16)	0.0204 (16)	0.0024 (13)	0.0028 (13)	−0.0043 (13)
C73	0.0184 (16)	0.0300 (19)	0.0202 (16)	0.0019 (14)	−0.0007 (13)	−0.0051 (13)
C43	0.0179 (16)	0.0276 (18)	0.0221 (17)	0.0054 (14)	0.0030 (13)	−0.0039 (14)
C47	0.0214 (16)	0.0223 (17)	0.0215 (16)	0.0090 (13)	0.0004 (13)	−0.0017 (13)
C16	0.0220 (16)	0.0161 (16)	0.0237 (17)	0.0016 (13)	0.0040 (13)	−0.0014 (12)
C4	0.0204 (16)	0.0196 (16)	0.0175 (15)	0.0001 (13)	−0.0005 (12)	−0.0024 (12)
C75	0.0248 (18)	0.029 (2)	0.0340 (19)	0.0036 (15)	0.0058 (15)	−0.0063 (15)
C64	0.0197 (16)	0.0250 (18)	0.0280 (18)	0.0034 (14)	0.0018 (13)	0.0016 (14)
C8	0.0206 (16)	0.0193 (16)	0.0246 (16)	0.0081 (13)	0.0016 (13)	−0.0018 (13)
C59	0.0158 (15)	0.0212 (16)	0.0213 (16)	0.0062 (13)	−0.0014 (12)	−0.0011 (13)
C22	0.0214 (17)	0.0158 (16)	0.0301 (18)	0.0043 (13)	−0.0005 (14)	0.0017 (13)
C36	0.0264 (18)	0.0192 (17)	0.0301 (18)	0.0053 (14)	−0.0002 (14)	−0.0031 (14)
C24	0.0226 (16)	0.0154 (16)	0.0222 (16)	0.0004 (13)	−0.0010 (13)	−0.0009 (12)
C65	0.0189 (16)	0.0251 (18)	0.0181 (16)	0.0003 (13)	0.0015 (12)	−0.0048 (13)
C45	0.0228 (17)	0.0233 (17)	0.0176 (15)	0.0014 (13)	−0.0001 (13)	−0.0022 (13)
C18	0.0210 (17)	0.038 (2)	0.0279 (18)	0.0074 (15)	0.0023 (14)	−0.0026 (16)
C69	0.0194 (16)	0.0218 (17)	0.0228 (16)	0.0044 (13)	−0.0003 (12)	−0.0050 (13)
C11	0.0255 (17)	0.0207 (17)	0.0163 (15)	0.0032 (13)	−0.0005 (13)	−0.0028 (12)
C25	0.0238 (17)	0.0187 (16)	0.0218 (17)	0.0055 (13)	−0.0028 (13)	−0.0004 (13)
C67	0.0196 (16)	0.0234 (17)	0.0239 (17)	0.0051 (13)	0.0012 (13)	−0.0030 (13)
C28	0.0213 (16)	0.0212 (17)	0.0229 (17)	0.0064 (14)	0.0017 (13)	−0.0021 (13)
C10	0.0201 (16)	0.0185 (16)	0.0229 (17)	0.0039 (13)	−0.0023 (13)	−0.0061 (13)
C2	0.0243 (16)	0.0174 (16)	0.0225 (16)	0.0077 (13)	−0.0009 (13)	−0.0006 (12)
C74	0.0211 (17)	0.0235 (18)	0.0308 (18)	0.0012 (14)	0.0036 (14)	−0.0028 (14)
C51	0.0175 (16)	0.0248 (17)	0.0192 (16)	0.0018 (13)	−0.0017 (12)	−0.0045 (13)
C1	0.0248 (17)	0.0195 (17)	0.0219 (16)	0.0047 (14)	−0.0015 (13)	−0.0012 (13)
C38	0.0252 (18)	0.0240 (18)	0.0274 (18)	−0.0007 (14)	0.0066 (14)	0.0003 (14)
C30	0.0178 (15)	0.0174 (16)	0.0223 (16)	0.0023 (12)	−0.0013 (12)	−0.0046 (12)
C78	0.0210 (17)	0.0212 (18)	0.0297 (18)	0.0029 (14)	0.0007 (14)	−0.0052 (14)
C61	0.0250 (18)	0.0298 (19)	0.0263 (18)	−0.0002 (15)	0.0008 (14)	0.0062 (14)
C29	0.0200 (16)	0.0220 (17)	0.0169 (15)	0.0012 (13)	−0.0007 (12)	−0.0025 (13)
C60	0.0209 (16)	0.0274 (18)	0.0187 (16)	0.0064 (14)	−0.0018 (13)	−0.0006 (13)
C41	0.0289 (19)	0.0271 (18)	0.0240 (17)	−0.0015 (15)	0.0045 (14)	−0.0040 (14)
C54	0.0183 (16)	0.0246 (18)	0.0311 (18)	0.0041 (14)	0.0036 (14)	−0.0048 (14)
C50	0.0205 (16)	0.0258 (18)	0.0175 (15)	0.0056 (13)	0.0007 (12)	−0.0020 (13)
C66	0.0189 (16)	0.0238 (17)	0.0202 (16)	0.0025 (13)	−0.0027 (12)	−0.0026 (13)
C9	0.0190 (16)	0.0220 (17)	0.0216 (16)	0.0013 (13)	−0.0015 (13)	−0.0018 (13)
C40	0.036 (2)	0.0211 (17)	0.0224 (17)	0.0044 (15)	0.0046 (15)	0.0032 (13)
C21	0.0225 (17)	0.0210 (17)	0.038 (2)	0.0045 (14)	0.0061 (15)	−0.0005 (15)
C58	0.0288 (18)	0.0223 (18)	0.0285 (18)	0.0059 (14)	0.0000 (14)	−0.0040 (14)
C33	0.0308 (19)	0.044 (2)	0.0239 (18)	0.0059 (17)	0.0039 (15)	−0.0037 (16)
C14	0.038 (2)	0.0162 (16)	0.0250 (18)	0.0048 (14)	0.0034 (15)	0.0006 (13)
C44	0.0205 (16)	0.0234 (17)	0.0208 (16)	0.0067 (13)	0.0022 (13)	−0.0056 (13)
C81	0.0241 (18)	0.0251 (19)	0.045 (2)	0.0022 (15)	−0.0006 (16)	0.0037 (16)
C77	0.0231 (18)	0.030 (2)	0.040 (2)	−0.0013 (15)	0.0053 (15)	0.0027 (16)
C42	0.0238 (17)	0.0247 (18)	0.0188 (16)	0.0035 (14)	0.0018 (13)	−0.0063 (13)
C13	0.0262 (18)	0.0212 (17)	0.0326 (19)	−0.0046 (14)	0.0064 (15)	−0.0034 (14)
C32	0.0212 (17)	0.032 (2)	0.0290 (18)	0.0043 (14)	0.0017 (14)	−0.0069 (15)
C55	0.0209 (17)	0.0177 (17)	0.046 (2)	0.0041 (13)	0.0032 (15)	0.0028 (15)
C39	0.0285 (18)	0.0241 (18)	0.0306 (19)	0.0090 (15)	0.0012 (15)	0.0013 (14)
C76	0.0187 (16)	0.044 (2)	0.0300 (19)	0.0001 (15)	0.0093 (14)	−0.0029 (16)
C35	0.0211 (18)	0.030 (2)	0.045 (2)	0.0027 (15)	0.0098 (16)	−0.0022 (16)
C15	0.0247 (17)	0.0222 (17)	0.0271 (18)	0.0094 (14)	−0.0003 (14)	−0.0001 (14)
C62	0.0242 (18)	0.038 (2)	0.0263 (18)	0.0062 (16)	0.0087 (14)	0.0010 (15)
C34	0.037 (2)	0.034 (2)	0.0293 (19)	0.0047 (16)	0.0155 (16)	0.0006 (15)
C56	0.0245 (18)	0.0292 (19)	0.036 (2)	0.0063 (15)	0.0068 (15)	0.0086 (15)
C19	0.034 (2)	0.044 (2)	0.0191 (17)	0.0087 (17)	0.0033 (14)	−0.0009 (15)
C57	0.035 (2)	0.0302 (19)	0.0262 (18)	0.0031 (16)	0.0038 (15)	−0.0011 (15)
C83	0.032 (2)	0.046 (2)	0.0235 (18)	−0.0090 (17)	0.0003 (15)	0.0016 (16)
C84	0.0222 (16)	0.0299 (19)	0.0282 (18)	−0.0025 (14)	0.0025 (14)	−0.0058 (15)
C82	0.028 (2)	0.042 (2)	0.033 (2)	−0.0046 (17)	−0.0042 (15)	0.0110 (17)
C20	0.0295 (19)	0.033 (2)	0.0285 (19)	0.0038 (16)	0.0115 (15)	0.0007 (15)
C12	0.0228 (17)	0.0215 (17)	0.0278 (18)	0.0019 (14)	−0.0006 (14)	−0.0040 (14)
Cl8	0.0284 (4)	0.0303 (5)	0.0336 (5)	0.0075 (4)	0.0059 (4)	−0.0005 (4)
Cl5	0.0323 (5)	0.0290 (5)	0.0348 (5)	0.0064 (4)	0.0081 (4)	0.0012 (4)
Cl12	0.0303 (5)	0.0274 (4)	0.0369 (5)	0.0039 (4)	0.0065 (4)	−0.0034 (4)
Cl3	0.0526 (6)	0.0589 (7)	0.0309 (5)	0.0177 (5)	−0.0004 (5)	−0.0042 (5)
Cl4	0.0617 (7)	0.0542 (7)	0.0312 (5)	0.0169 (6)	0.0012 (5)	−0.0016 (5)
Cl10	0.0671 (8)	0.0633 (8)	0.0297 (5)	0.0260 (6)	0.0004 (5)	−0.0062 (5)
Cl11	0.0380 (6)	0.0282 (5)	0.0789 (8)	0.0028 (4)	−0.0071 (5)	−0.0001 (5)
Cl9	0.0672 (8)	0.0572 (7)	0.0292 (5)	0.0254 (6)	0.0032 (5)	−0.0019 (5)
Cl7	0.0368 (6)	0.0286 (5)	0.0854 (9)	0.0058 (4)	−0.0036 (6)	−0.0016 (5)
C87	0.032 (2)	0.0295 (19)	0.0300 (19)	0.0103 (16)	−0.0002 (15)	0.0013 (15)
C88	0.0272 (19)	0.0308 (19)	0.033 (2)	0.0077 (16)	−0.0043 (15)	−0.0030 (16)
Cl1	0.0266 (5)	0.0371 (6)	0.0780 (8)	0.0024 (4)	0.0067 (5)	−0.0134 (5)
Cl6	0.0304 (5)	0.0353 (6)	0.1000 (10)	0.0036 (4)	0.0084 (6)	−0.0069 (6)
C86	0.0301 (19)	0.031 (2)	0.039 (2)	0.0061 (16)	0.0042 (16)	−0.0035 (16)
C85	0.030 (2)	0.031 (2)	0.036 (2)	0.0109 (16)	0.0018 (16)	−0.0056 (16)
Cl2	0.0274 (4)	0.0296 (5)	0.0350 (5)	0.0050 (4)	0.0044 (4)	−0.0001 (4)

### Geometric parameters (Å, °)

**Table:**

S5—C45	1.745 (4)	C59—C51	1.480 (5)
S5—C43	1.753 (3)	C22—C21	1.392 (5)
S4—C24	1.743 (3)	C22—H22	0.9300
S4—C2	1.759 (4)	C36—C35	1.377 (5)
S8—C66	1.744 (4)	C36—H36	0.9300
S8—C44	1.754 (3)	C24—C25	1.370 (5)
S1—C1	1.748 (4)	C65—C66	1.435 (5)
S1—C3	1.752 (3)	C45—C50	1.373 (5)
S7—C65	1.748 (3)	C18—C19	1.372 (5)
S7—C44	1.755 (4)	C18—H18	0.9300
S3—C23	1.748 (3)	C11—C12	1.388 (5)
S3—C2	1.756 (3)	C11—C10	1.484 (5)
S6—C46	1.751 (3)	C25—H25	0.9300
S6—C43	1.758 (4)	C67—C66	1.365 (5)
S2—C4	1.749 (3)	C67—H67	0.9300
S2—C1	1.758 (3)	C28—H28	0.9300
C68—N8	1.358 (4)	C10—C9	1.447 (5)
C68—C69	1.409 (5)	C2—C1	1.341 (5)
C68—C67	1.415 (5)	C74—H74	0.9300
N7—C71	1.319 (4)	C38—C39	1.370 (5)
N7—C69	1.367 (4)	C38—H38	0.9300
N3—C29	1.312 (4)	C30—C29	1.447 (5)
N3—C27	1.358 (4)	C78—C77	1.377 (5)
C17—C22	1.389 (5)	C78—H78	0.9300
C17—C18	1.396 (5)	C61—C60	1.386 (5)
C17—C9	1.486 (5)	C61—C62	1.386 (5)
C31—C32	1.382 (5)	C61—H61	0.9300
C31—C36	1.406 (5)	C60—H60	0.9300
C31—C30	1.477 (5)	C41—C42	1.383 (5)
N6—C52	1.322 (4)	C41—C40	1.387 (5)
N6—C48	1.361 (4)	C41—H41	0.9300
C26—N4	1.360 (4)	C54—C55	1.380 (5)
C26—C25	1.407 (5)	C54—H54	0.9300
C26—C27	1.417 (5)	C50—H50	0.9300
N2—C10	1.321 (5)	C40—C39	1.391 (5)
N2—C6	1.358 (4)	C40—H40	0.9300
C7—N1	1.356 (4)	C21—C20	1.387 (5)
C7—C8	1.410 (5)	C21—H21	0.9300
C7—C6	1.428 (5)	C58—C57	1.377 (5)
N4—C30	1.318 (4)	C58—H58	0.9300
C80—C79	1.383 (5)	C33—C32	1.371 (5)
C80—C81	1.391 (5)	C33—C34	1.384 (6)
C80—H80	0.9300	C33—H33	0.9300
N5—C51	1.316 (4)	C14—C13	1.380 (5)
N5—C49	1.368 (4)	C14—C15	1.382 (5)
C49—C50	1.412 (5)	C14—H14	0.9300
C49—C48	1.419 (5)	C81—C82	1.387 (6)
C27—C28	1.422 (5)	C81—H81	0.9300
C3—C8	1.371 (5)	C77—C76	1.392 (6)
C3—C4	1.419 (5)	C77—H77	0.9300
C72—N8	1.335 (5)	C42—H42	0.9300
C72—C71	1.433 (5)	C13—C12	1.386 (5)
C72—C73	1.493 (5)	C13—H13	0.9300
C53—C58	1.397 (5)	C32—H32	0.9300
C53—C54	1.402 (5)	C55—C56	1.370 (6)
C53—C52	1.482 (5)	C55—H55	0.9300
C70—C65	1.370 (5)	C39—H39	0.9300
C70—C69	1.402 (5)	C76—H76	0.9300
C70—H70	0.9300	C35—C34	1.377 (6)
C79—C84	1.391 (5)	C35—H35	0.9300
C79—C71	1.488 (5)	C15—H15	0.9300
N1—C9	1.309 (4)	C62—H62	0.9300
C48—C47	1.403 (5)	C34—H34	0.9300
C5—C4	1.367 (5)	C56—C57	1.392 (5)
C5—C6	1.411 (5)	C56—H56	0.9300
C5—H5	0.9300	C19—C20	1.384 (6)
C23—C28	1.367 (5)	C19—H19	0.9300
C23—C24	1.426 (5)	C57—H57	0.9300
C52—C51	1.443 (5)	C83—C82	1.376 (6)
C46—C47	1.365 (5)	C83—C84	1.395 (5)
C46—C45	1.422 (5)	C83—H83	0.9300
C63—C62	1.376 (6)	C84—H84	0.9300
C63—C64	1.381 (5)	C82—H82	0.9300
C63—H63	0.9300	C20—H20	0.9300
C37—C42	1.396 (5)	C12—H12	0.9300
C37—C38	1.397 (5)	Cl8—C87	1.764 (4)
C37—C29	1.486 (5)	Cl5—C86	1.765 (4)
C73—C74	1.393 (5)	Cl12—C88	1.762 (4)
C73—C78	1.397 (5)	Cl3—C85	1.749 (4)
C43—C44	1.338 (5)	Cl4—C86	1.759 (4)
C47—H47	0.9300	Cl10—C88	1.755 (4)
C16—C15	1.396 (5)	Cl11—C88	1.760 (4)
C16—C11	1.398 (5)	Cl9—C87	1.756 (4)
C16—H16	0.9300	Cl7—C87	1.755 (4)
C75—C74	1.377 (5)	C87—H87	0.9800
C75—C76	1.381 (6)	C88—H88	0.9800
C75—H75	0.9300	Cl1—C85	1.771 (4)
C64—C59	1.407 (5)	Cl6—C86	1.765 (4)
C64—H64	0.9300	C86—H86	0.9800
C8—H8	0.9300	C85—Cl2	1.770 (4)
C59—C60	1.391 (5)	C85—H85	0.9800
			
C45—S5—C43	96.02 (17)	C1—C2—S4	122.5 (2)
C24—S4—C2	95.88 (16)	S3—C2—S4	115.43 (19)
C66—S8—C44	96.17 (16)	C75—C74—C73	120.8 (3)
C1—S1—C3	95.57 (16)	C75—C74—H74	119.6
C65—S7—C44	95.98 (16)	C73—C74—H74	119.6
C23—S3—C2	96.00 (16)	N5—C51—C52	120.7 (3)
C46—S6—C43	95.76 (16)	N5—C51—C59	117.0 (3)
C4—S2—C1	95.70 (16)	C52—C51—C59	122.3 (3)
N8—C68—C69	121.7 (3)	C2—C1—S1	122.7 (2)
N8—C68—C67	119.1 (3)	C2—C1—S2	121.3 (2)
C69—C68—C67	119.2 (3)	S1—C1—S2	115.9 (2)
C71—N7—C69	117.7 (3)	C39—C38—C37	120.1 (3)
C29—N3—C27	118.3 (3)	C39—C38—H38	119.9
C22—C17—C18	119.0 (3)	C37—C38—H38	119.9
C22—C17—C9	120.2 (3)	N4—C30—C29	121.0 (3)
C18—C17—C9	120.8 (3)	N4—C30—C31	116.4 (3)
C32—C31—C36	118.4 (3)	C29—C30—C31	122.4 (3)
C32—C31—C30	121.1 (3)	C77—C78—C73	120.9 (3)
C36—C31—C30	120.4 (3)	C77—C78—H78	119.6
C52—N6—C48	118.7 (3)	C73—C78—H78	119.6
N4—C26—C25	119.6 (3)	C60—C61—C62	120.6 (3)
N4—C26—C27	120.3 (3)	C60—C61—H61	119.7
C25—C26—C27	120.0 (3)	C62—C61—H61	119.7
C10—N2—C6	118.2 (3)	N3—C29—C30	120.9 (3)
N1—C7—C8	119.9 (3)	N3—C29—C37	116.4 (3)
N1—C7—C6	120.2 (3)	C30—C29—C37	122.7 (3)
C8—C7—C6	119.8 (3)	C61—C60—C59	119.9 (3)
C30—N4—C26	118.2 (3)	C61—C60—H60	120.0
C79—C80—C81	120.5 (3)	C59—C60—H60	120.0
C79—C80—H80	119.7	C42—C41—C40	119.6 (3)
C81—C80—H80	119.7	C42—C41—H41	120.2
C51—N5—C49	118.5 (3)	C40—C41—H41	120.2
N5—C49—C50	119.5 (3)	C55—C54—C53	120.1 (3)
N5—C49—C48	120.7 (3)	C55—C54—H54	120.0
C50—C49—C48	119.8 (3)	C53—C54—H54	120.0
N3—C27—C26	121.0 (3)	C45—C50—C49	119.0 (3)
N3—C27—C28	119.5 (3)	C45—C50—H50	120.5
C26—C27—C28	119.5 (3)	C49—C50—H50	120.5
C8—C3—C4	121.1 (3)	C67—C66—C65	120.2 (3)
C8—C3—S1	122.3 (3)	C67—C66—S8	123.8 (3)
C4—C3—S1	116.6 (3)	C65—C66—S8	116.0 (2)
N8—C72—C71	121.2 (3)	N1—C9—C10	121.4 (3)
N8—C72—C73	115.5 (3)	N1—C9—C17	115.9 (3)
C71—C72—C73	123.3 (3)	C10—C9—C17	122.6 (3)
C58—C53—C54	118.5 (3)	C41—C40—C39	119.4 (3)
C58—C53—C52	120.9 (3)	C41—C40—H40	120.3
C54—C53—C52	120.5 (3)	C39—C40—H40	120.3
C72—N8—C68	117.1 (3)	C20—C21—C22	120.3 (3)
C65—C70—C69	119.3 (3)	C20—C21—H21	119.8
C65—C70—H70	120.4	C22—C21—H21	119.8
C69—C70—H70	120.4	C57—C58—C53	120.6 (3)
C80—C79—C84	119.6 (3)	C57—C58—H58	119.7
C80—C79—C71	118.7 (3)	C53—C58—H58	119.7
C84—C79—C71	121.6 (3)	C32—C33—C34	120.1 (4)
C9—N1—C7	118.5 (3)	C32—C33—H33	119.9
N6—C48—C47	119.8 (3)	C34—C33—H33	119.9
N6—C48—C49	120.0 (3)	C13—C14—C15	120.1 (3)
C47—C48—C49	120.1 (3)	C13—C14—H14	119.9
C4—C5—C6	119.7 (3)	C15—C14—H14	119.9
C4—C5—H5	120.1	C43—C44—S8	122.1 (2)
C6—C5—H5	120.1	C43—C44—S7	122.3 (2)
C28—C23—C24	121.1 (3)	S8—C44—S7	115.6 (2)
C28—C23—S3	122.9 (3)	C82—C81—C80	119.8 (4)
C24—C23—S3	116.0 (3)	C82—C81—H81	120.1
N2—C6—C5	119.8 (3)	C80—C81—H81	120.1
N2—C6—C7	120.7 (3)	C78—C77—C76	119.6 (3)
C5—C6—C7	119.5 (3)	C78—C77—H77	120.2
N6—C52—C51	121.0 (3)	C76—C77—H77	120.2
N6—C52—C53	115.5 (3)	C41—C42—C37	121.0 (3)
C51—C52—C53	123.3 (3)	C41—C42—H42	119.5
C47—C46—C45	120.8 (3)	C37—C42—H42	119.5
C47—C46—S6	123.0 (3)	C14—C13—C12	120.3 (3)
C45—C46—S6	116.3 (3)	C14—C13—H13	119.9
N7—C71—C72	121.6 (3)	C12—C13—H13	119.9
N7—C71—C79	115.0 (3)	C33—C32—C31	121.2 (3)
C72—C71—C79	123.4 (3)	C33—C32—H32	119.4
C62—C63—C64	120.5 (3)	C31—C32—H32	119.4
C62—C63—H63	119.8	C56—C55—C54	121.1 (3)
C64—C63—H63	119.8	C56—C55—H55	119.4
C42—C37—C38	118.7 (3)	C54—C55—H55	119.4
C42—C37—C29	121.3 (3)	C38—C39—C40	121.1 (3)
C38—C37—C29	120.0 (3)	C38—C39—H39	119.5
C74—C73—C78	118.5 (3)	C40—C39—H39	119.5
C74—C73—C72	120.2 (3)	C75—C76—C77	120.2 (3)
C78—C73—C72	121.3 (3)	C75—C76—H76	119.9
C44—C43—S5	122.6 (2)	C77—C76—H76	119.9
C44—C43—S6	121.9 (2)	C34—C35—C36	120.5 (3)
S5—C43—S6	115.58 (19)	C34—C35—H35	119.8
C46—C47—C48	119.4 (3)	C36—C35—H35	119.8
C46—C47—H47	120.3	C14—C15—C16	120.1 (3)
C48—C47—H47	120.3	C14—C15—H15	119.9
C15—C16—C11	119.6 (3)	C16—C15—H15	119.9
C15—C16—H16	120.2	C63—C62—C61	119.8 (3)
C11—C16—H16	120.2	C63—C62—H62	120.1
C5—C4—C3	120.6 (3)	C61—C62—H62	120.1
C5—C4—S2	123.4 (3)	C35—C34—C33	119.7 (3)
C3—C4—S2	116.1 (3)	C35—C34—H34	120.2
C74—C75—C76	120.0 (4)	C33—C34—H34	120.2
C74—C75—H75	120.0	C55—C56—C57	119.4 (3)
C76—C75—H75	120.0	C55—C56—H56	120.3
C63—C64—C59	120.1 (3)	C57—C56—H56	120.3
C63—C64—H64	119.9	C18—C19—C20	120.7 (3)
C59—C64—H64	119.9	C18—C19—H19	119.7
C3—C8—C7	119.2 (3)	C20—C19—H19	119.7
C3—C8—H8	120.4	C58—C57—C56	120.3 (4)
C7—C8—H8	120.4	C58—C57—H57	119.8
C60—C59—C64	119.0 (3)	C56—C57—H57	119.8
C60—C59—C51	121.6 (3)	C82—C83—C84	120.7 (4)
C64—C59—C51	119.3 (3)	C82—C83—H83	119.7
C17—C22—C21	120.1 (3)	C84—C83—H83	119.7
C17—C22—H22	120.0	C79—C84—C83	119.5 (4)
C21—C22—H22	120.0	C79—C84—H84	120.2
C35—C36—C31	120.1 (3)	C83—C84—H84	120.2
C35—C36—H36	120.0	C83—C82—C81	119.8 (4)
C31—C36—H36	120.0	C83—C82—H82	120.1
C25—C24—C23	120.3 (3)	C81—C82—H82	120.1
C25—C24—S4	123.1 (3)	C19—C20—C21	119.3 (3)
C23—C24—S4	116.5 (3)	C19—C20—H20	120.3
C70—C65—C66	120.5 (3)	C21—C20—H20	120.3
C70—C65—S7	123.3 (3)	C13—C12—C11	120.2 (3)
C66—C65—S7	116.1 (3)	C13—C12—H12	119.9
C50—C45—C46	120.8 (3)	C11—C12—H12	119.9
C50—C45—S5	122.8 (3)	Cl7—C87—Cl9	110.8 (2)
C46—C45—S5	116.3 (3)	Cl7—C87—Cl8	110.2 (2)
C19—C18—C17	120.6 (3)	Cl9—C87—Cl8	110.9 (2)
C19—C18—H18	119.7	Cl7—C87—H87	108.3
C17—C18—H18	119.7	Cl9—C87—H87	108.3
N7—C69—C70	118.6 (3)	Cl8—C87—H87	108.3
N7—C69—C68	120.5 (3)	Cl10—C88—Cl11	110.6 (2)
C70—C69—C68	120.8 (3)	Cl10—C88—Cl12	110.5 (2)
C12—C11—C16	119.6 (3)	Cl11—C88—Cl12	110.2 (2)
C12—C11—C10	121.5 (3)	Cl10—C88—H88	108.5
C16—C11—C10	118.9 (3)	Cl11—C88—H88	108.5
C24—C25—C26	119.8 (3)	Cl12—C88—H88	108.5
C24—C25—H25	120.1	Cl4—C86—Cl5	110.6 (2)
C26—C25—H25	120.1	Cl4—C86—Cl6	110.6 (2)
C66—C67—C68	119.9 (3)	Cl5—C86—Cl6	109.8 (2)
C66—C67—H67	120.0	Cl4—C86—H86	108.6
C68—C67—H67	120.0	Cl5—C86—H86	108.6
C23—C28—C27	119.2 (3)	Cl6—C86—H86	108.6
C23—C28—H28	120.4	Cl3—C85—Cl2	110.8 (2)
C27—C28—H28	120.4	Cl3—C85—Cl1	111.0 (2)
N2—C10—C9	120.7 (3)	Cl2—C85—Cl1	109.3 (2)
N2—C10—C11	116.1 (3)	Cl3—C85—H85	108.6
C9—C10—C11	123.2 (3)	Cl2—C85—H85	108.6
C1—C2—S3	122.1 (2)	Cl1—C85—H85	108.6

### Hydrogen-bond geometry (Å, °)

**Table:**

*D*—H···*A*	*D*—H	H···*A*	*D*···*A*	*D*—H···*A*
C8—H8···Cl2^i^	0.93	2.94	3.676 (4)	137.
C85—H85···N2^ii^	0.98	2.31	3.233 (5)	156.
C12—H12···N6^iii^	0.93	2.61	3.344 (4)	136.
C86—H86···N3^iv^	0.98	2.29	3.223 (5)	158.
C88—H88···N8^iv^	0.98	2.28	3.199 (5)	155.
C87—H87···N5^v^	0.98	2.32	3.246 (5)	157.
C60—H60···N1^vi^	0.93	2.63	3.392 (5)	139.
C78—H78···N4^vii^	0.93	2.62	3.427 (4)	145.
C42—H42···N7^v^	0.93	2.61	3.358 (4)	138.

Symmetry codes: (i) *x*−1, *y*, *z*; (ii) *x*+1/2, *y*+1/2, *z*; (iii) *x*, *y*−1, *z*; (iv) *x*−1/2, *y*−1/2, *z*; (v) *x*+1/2, *y*−1/2, *z*; (vi) *x*−1/2, *y*+1/2, *z*; (vii) *x*, *y*+1, *z*.
